# Elevated Microbially-Derived Metabolites in Autism: A Possible Diagnostic Screening Test for a Distinct ASD Phenotype

**DOI:** 10.21203/rs.3.rs-7643826/v1

**Published:** 2025-10-02

**Authors:** Christina Flynn, Kevin Carr, Paul Whiteley, Khemlal Nirmalkar, Andrew Bellinghiere, Juergen Hahn, Hongbin Liu, Halil Arici, Laura Hewitson, Morgan Devlin, Elena Pollard, Khyatiben Pathak, Krystine Garcia, Anakaren Rosales, Patrick Pirrotte, Daniel Kalb, Rebekah Keen, Victoria Kenyon, Alessio Fasano, Rosa Krajmalnik-Brown, James Adams

**Affiliations:** Arizona State University; Rensselaer Polytechnic Institute; Massachusetts General Hospital; Arizona State University; ASU

**Keywords:** Autism Spectrum Disorder, Diagnostics, Biomarker, Phenotype, Metabolomics, microbial metabolites

## Abstract

Many studies have confirmed that a subset of children with autism spectrum disorder (ASD) have unusually high urinary concentrations of harmful microbially-derived metabolites (MDMs) such as p-cresol sulfate and indoxyl sulfate. We hypothesized that these MDMs may affect neurodevelopment through the gut-brain axis and that a sub-phenotype characterized by gut dysbiosis may be present in most ASD individuals. This multi-site study involved measuring the concentrations of many MDMs in the urine of 52 children with ASD and 47 healthy, typically developing (TD) children, aged 2 to 11 years. The measurements were conducted first with untargeted semiquantitative Liquid Chromatography and Mass Spectrometry (LC-MS), followed by targeted quantitative LC-MS. The ASD group had significantly higher concentrations of many MDMs compared to the TD group. The MDMs included phenylalanine-derived, tryptophan-derived, and yeast-derived MDMs. Almost all children with ASD had one or more MDMs at concentrations above any TD child, and sometimes 100–1000x higher. The children with ASD had an average of 3 MDMs at levels above any TD child, compared to zero (by definition) for the TD children. Classification using one or more elevated MDM yielded a sensitivity of 90% and a specificity of 100%. This MDM System^™^ is a promising non-invasive method for diagnostic screening for ASD in children ages 2 to 11 years. These data also suggest approximately 90% of children with ASD have a distinct phenotype of ASD, which we propose naming ASD associated with Microbially-Derived Metabolites (ASD-MDM), defined by quantitative laboratory measurements of these metabolites in urine.

## Introduction

Autism Spectrum Disorder (ASD) is a label for a collection of symptoms that begin in early childhood and involve major but variable impairments in social communication and stereotypical or repetitive behaviors^1^. ASD prevalence has dramatically increased from approximately 1 in 10,000 in 1980 to 323 per 10,000 in 2022 in the United States^2,3^. Average lifetime care costs are estimated at $3.6 million per individual; however, individuals severely affected by the disorder can require much more expensive care^4^.

Early behavioral intervention can reduce the core symptoms of ASD and improve outcomes in later life^5,6^. Behavioral interventions are most effective beginning in early infancy, preferably in the first 24 months of life^7^; however, the average age of first diagnostic assessment interview in the United States (US) is 47 months^8^. Thus, there exists an urgent need to develop a non-invasive screening tool to identify those at high risk for ASD to facilitate earlier diagnosis and treatment ^9,10^. Identifying children with ASD as young as possible and treating them can improve long-term outcomes, enhance quality of life, and potentially save hundreds of billions of dollars per year^11^.

ASD is thought to result from a complex interplay of genetic and environmental factors^12^. Different factors are important for different people, with about 10% of cases involving major genetic disorders such as Fragile X, and about 90% of cases being of unknown origin. Furthermore, stratifying the heterogeneous ASD population into distinct, well-defined phenotypes will likely improve treatment, as targeted therapeutic plans can be developed for each phenotype.

ASD involves many co-occurring symptoms. One major subgroup involveschronic gastrointestinal (GI) symptoms, which affect approximately 40% of individuals with ASD^13,14^. The intensity of these GI symptoms - including constipation, diarrhea, gas, bloating, abdominal pains, and/or reflux – seemingly correlates with the severity of ASD traits^15,16^. Research suggests that the GI symptoms begin in the first three years of life^17^, corresponding to a similar timeframe as the development of ASD symptoms. The GI symptoms appear to primarily be due to a disruption of the human commensal gut microbiota, as treatment with Microbiota Transplant Therapy^15,16,18^ and similar methods have demonstrated substantial improvement in GI and ASD symptoms^19,20^. Similarly, a meta-analysis of many studies found that most children with ASD have gut dysbiosis, including over 500 species that are present at much higher relative abundance and over 100 species present at much lower relative abundance compared to typically developing (TD) children^21^.

Dysbiosis in ASD has been shown to influence the concentrations of short-chain fatty acids (SCFAs), proinflammatory cytokines, xenobiotic detoxification pathways, and neurotransmitter regulation^22,23^. Based on these findings, we hypothesize that a disrupted microbiome may influence neurodevelopment via the gut-brain axis and that a distinct ASD phenotype involving gut dysbiosis may exist.

A critical way in which gut microbiota affects ASD symptoms is by producing metabolites which, by themselves, or through human metabolism, are harmful at high concentrations^24–32^. One hallmark example is *p*-cresol, which is produced from phenylalanine or tyrosine by over 60 gut bacteria^33,34^. *P*-cresol is subsequently processed in the intestine and liver into *p*-cresol sulfate (pCS), which is higher in the urine of children with autism vs. controls in 17 of 17 studies^1,35–49^. *P*-cresol and pCS have many adverse effects on the gut, mitochondria, brain, kidneys, and immune syste^50^. *P*-cresol causes autistic symptoms when administered to animals, and fecal transplant from healthy animals restores normal function^25,51^. Similarly, urinary concentrations of *p*-cresol are correlated with the severity of ASD symptoms in humans^35,36^. Thus, it seems likely that p-cresol may contribute to ASD symptoms in humans.

P-cresol is just one of many harmful microbial metabolites found to be significantly higher in children with ASD. Another harmful microbial metabolite found at higher concentrations in ASD is indoxyl sulfate. Further information about the adverse effects of this compound is summarized by Hill et al., 2024^52^. Table 1 lists microbially related metabolites that have been consistently reported as elevated in ASD. Elevated levels of hippuric and hydroxybenzoic acids demonstrate an overstressed detoxification metabolism, potentially secondary to sulfate metabolism dysfunction caused by the production of p-cresol sulfate and indoxyl sulfate. Among microbial metabolites, yeast-derived metabolites have also been associated with autism in 20%–30% of the population^53^. The yeast metabolites (arabinitol, citramalic acid, tricarballylic acid) are not clearly toxic but are biomarkers of high levels of yeast/fungi, which produce other harmful toxins^54–56^.

This study aimed to develop a biomedical diagnostic screening test for ASD. We compared concentrations of MDMs in the urine of children with ASD and typically developing children, first using untargeted metabolomics and then targeted metabolomics. A novel multivariate analysis called the Microbially-Derived Metabolite System^™^, or “The MDM System^™^, was developed as a diagnostic screening tool to identify the subset of children with ASD who have intestinal dysbiosis.

## Methods

### Participants:

This study, approved by the Institutional Review Board of Arizona State University (STUDY00008750), and the Institutional Review Board of Harvard Medical School, was advertised to autism families and parents of TD children at four locations: Arizona (Arizona State University), Massachusetts (Harvard Medical School), Tennessee (Cool Springs Family Medicine), and Texas (The Johnson Medical Center for Child Health and Development). All participants met with a study coordinator to review and sign the consent form. Children over the age of 7 also signed an assent form if they were developmentally/cognitively able. The study overall design is shown in Figure A in the supplement. All 52 children affected by ASD and 47 children who were healthy and typically developing (TD) included in the study were between 2–11 years old (See Table 3 below for detailed information about participant characteristics).

The inclusion and exclusion criteria are shown.

#### Autism Spectrum Disorder (ASD) Inclusion Criteria

- Diagnosis of ASD by a psychiatrist, developmental pediatrician, or similar professional, and verification by the Childhood Autism Rating Scale (CARS-2)
- Age 2–11 years
- Parents are fluent in English.

#### ASD Exclusion Criteria

- Major single-gene disorder such as Fragile X
- An identified major brain abnormality
- Current participation in a treatment study, or major change in medications, supplements, diet, or therapies in the last 2 months
- Bacterial or viral illness at time of urine sample collection (temporary exclusion; delay until healthy)

#### Typically Developing Inclusion Criteria

- Age 2–11 years
- Good physical and mental health
- SRS score in the typical range (SRS raw score < 60)
- Parents are fluent in English, and the child speaks English

#### Typically Developing Exclusion Criteria

- Sibling or parent with ASD
- Requires special services in school, or academic ability well below grade level.
- A major single-gene disorder such as Fragile X
- Major brain abnormality
- Major physical or mental health problem
- Current participation in a treatment study, or major change in medications, supplements, diet, or therapies in the last 2 months
- Bacterial or viral illness at the time of urine sample collection (temporary exclusion; delay until healthy)

### Questionnaires:

Parents of ASD children were required to provide evidence of a previous diagnosis of ASD for their child. This was confirmed by our expert autism assessor conducting an evaluation of the child and a discussion with their parent/guardian using the Childhood Autism Rating Scale (CARS). Parents of the ASD and TD children also completed a medical history form, the Social Responsiveness Scale-2 (SRS-2), and the Parental Global Impressions of Autism (PGIA). The SRS form was also used to confirm ASD diagnosis (raw SRS score above 68), and to ensure that the TD children did not present with clinically relevant autistic features.

### Sample Collection:

Urine samples were collected from study participants. Most samples were a first-morning sample, but some children were not dry overnight, so the urine sample collected was a “spot” urine sample in the early morning. Urine samples were stored in the family’s freezer and transferred for storage in −80°C freezers until prepared for analysis. For the semiquantitative analysis (using 50 ASDs, 47 TDs) and quantitative analyses (using 52 ASDs, 47 TDs), the two datasets are slightly different because five participants did not overlap due to several factors, including lost samples, shipping errors, and sample arrival dates.

### Metabolomics Measurements

**Figure 1:**
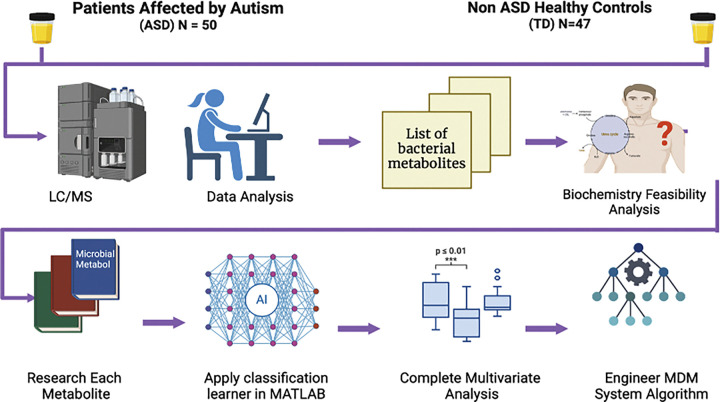
Overview of the study process to create the MDM System^™^. Untargeted metabolomics was undertaken on 50 patients affected by ASD and 47 TD individuals via LC/MS. Univariate analysis discovered 40 microbially derived metabolites with a raw p-value < 0.05. Each metabolite was evaluated based on biochemistry and literature review. The data was compiled into MATLAB software or multivariate analysis, leading to the development of the MDM System.

To develop a diagnostic screening test for ASD, this project was divided into two parts: an initial untargeted analysis followed by a targeted analysis. Untargeted semiquantitative metabolomics was first conducted by the Translational Research Institute of Genomics (TGEN, USA). Potential metabolites of interest were identified, and a separate independent laboratory (Analutos Ltd, UK) validated the analytes using quantitative measurements with commercial standards (Table 2). Figure 1 shows the overall work process and flow used to develop the MDM system.

For untargeted metabolomics, metabolite extraction was performed by adding 3-fold excess of acetonitrile: methanol (3:1, v/v) to the urine for HILIC and for reversed phase chromatography, urine was diluted with equal volume of water. The metabolite extracts were clarified by centrifugation at 13,226 ×*g* at 4°C for 15 min. The resulting supernatant were subjected to liquid chromatography and mass spectrometry analysis using Orbitrap Fusion Lumos Tribrid mass spectrometer and raw data were analyzed on Compound Discoverer 3.2 (Thermo Fisher Scientific, Ca) for compound annotation and relative quantitation as described previously ^67,68^.The MS/MS spectrum for each annotated compounds were reviewed for verifying compound identity. Relative abundances of metabolites were normalized using urinary creatinine to account for differences in hydration. Specific gravity was measured using hand-held refractometer and creatinine was measured using Jaffe method. Targeted quantitative analysis was performed by an independent commercial research laboratory,

Analutos (analutos.com, U.K) performed the quantitative portion of the analysis. (See table ) Briefly, urine samples were thawed at 5 degrees C and precipitates allowed to settle until thawed. 50ul of each sample was diluted with 100ul of water and 20ul of internal standard solution. Compounds were separated using reverse-phase High Performance Liquid Chromatography using a 10 ml water and 10 ml methanol solution, then modified with ammonium formate for the mobile phase. Analysis was performed using an Agilent—6530a-Q-ToF in negative mode. All Agilent operating instructions were followed per the operations manual. Creatinine was also measured in the same way.

Relative peak intensities were determined for each participant for each metabolite. Creatinine was also measured to use for normalization of metabolite concentrations to account for differences in hydration. The metabolites chosen for evaluation were mostly formed by microbial modification of tryptophan, phenylalanine, or tyrosine, and then, in some cases, also later modified by human metabolism. The remaining compounds were a) generated from yeasts or b) had been previously associated with dysbiosis. One such compound is Arabinitol, as high concentrations in urine indicate overgrowth of yeast within the gut (see Table 1). For each metabolite, a relative reference range was created by determining 0%–100% of the TD range. There is no known pathway for humans to produce any of these metabolites, and there is no significant dietary source known for them.

### Statistical Analysis

Univariate analyses were performed on both investigative datasets using Welch’s t-test. Since most metabolites had a subset of ASD participants’ concentrations above 100% of the TD range, the data were square root-transformed to determine normality, and a t-test was also conducted on the transformed data. False discovery rate adjustment was also performed on the semiquantitative analysis to minimize false positives (q < 0.05), which is the minimum number of metabolites that were expected to be significantly different by random chance using a p-value < 0.05. The Semiquantitative nature of untargeted LC/MS measurements reports peak intensities rather than definitive concentrations, so peak intensity averages for each group, percent difference between the two groups, individual AUROC, and p-value for each metabolite are listed in the tables below. One significant limitation of untargeted LC/MS measurements is that metabolites can be misidentified. This does not apply to quantitative results, as their experiment utilized commercially available standards to determine concentrations and identities of metabolites.

Multivariate Analysis –The MDM System^™^: A novel multivariate scoring method called the Microbially-Derived Metabolite System (MDM System^™^) was developed for analyzing the metabolite data, based on the hypothesis that an extremely high peak intensity of one or more MDMs might be sufficient to classify a person with gut dysbiosis associated with ASD. For each participant, the relative peak intensity (or concentration) of each metabolite (normed by creatinine) was compared to the TD reference range. If higher, a point was added to that participant’s individual MDM Total Score, and if not, no point was awarded. All TD participants received a score of zero, since all were within the TD reference range. Thus, the total MDM score represents the number of MDMs that were unusually high (above that of any TD child).

Additional Multivariate Analysis. Standard multivariate statistical analysis was performed in MATLAB, using Fisher Discriminant analysis (FDA), neural networks, and Naïve Bayes methods, which also found high sensitivity and specificity – see the Supplement for details.

## Results

### Four Study Sites in Four States Uncovered Similar Participants Characteristics

52 children with ASD and 47 TD children were recruited at four different study sites across the United States (Arizona, Texas, Tennessee, Massachusetts) to provide geographic diversity. Participant characteristics are listed in Table 3 below. All ASD participants had a clinical diagnosis of ASD based on DSM-5 criteria. The diagnoses were also confirmed in 51 of the 52 children with ASD with CARS scores greater than 28.5–30, depending on age and functionality. One child was not evaluated by CARS but had an SRS-2 raw score of 100 and PGIA of 2.8 (both values well above the cut-offs of 68 and 1.5, respectively, for ASD, so they were also included. All TD children had SRS-2 raw scores below 60, and PGIA scores below 1.5.

Age ranges of ASD and TD participants were closely matched. The ASD group had a higher fraction of males, but the TD group was purposely chosen to have a balance of males and females to establish a reliable reference range for both genders. Analyses were performed comparing total MDM scores separately for males and females and found no significant differences. As expected, significant differences were found between SRS-2 scores and PGIA scores between the ASD group and the TD group. Two ASD children were included in the quantitative analysis but were not included in the semiquantitative analysis due to sample availability issues.

### Univariate Semi-Quantitative Analysis Found Many MDMs Significantly Higher in ASD than Controls

The metabolites chosen for this analysis were investigated because of they are microbially produced. Results are displayed in Table 4, sorted by their origin (phenylalanine-derived, tryptophan-derived, or yeast/other). The analysis was based on LC-MS peak intensities normalized by creatinine. Six phenylalanine-derived microbial metabolites were significantly higher in the ASD group than the TD group, with the average difference ranging from 29% higher to 228% higher, and the percentage of individuals in the ASD group above the highest TD peak intensity ranged from 10% to 32%. Eight tryptophan-derived microbial metabolites were significantly higher in the ASD group than the TD group, with the difference ranging from 38% higher to 1,882% higher, and the percentage of individuals in the ASD group above the highest TD peak intensity ranged from 10% to 44%. Concentrations of arabinitol, a yeast metabolite, were significantly higher in the ASD group (51% higher, p = 0.002), with 17% of children with ASD having concentrations above that of the highest TD case. N-Formyl methionine was 70% lower in the ASD group.

The average percent difference in peak intensities (normalized by creatinine) between ASD and TD, the AUROC of each metabolite, the p-value, and the q-value are displayed for each metabolite. All results are statistically significantly different (p < 0.05).

Most ASD participants had very elevated peak intensities (above that of any TD child) of one or more tryptophan metabolites (64%), phenylalanine-derived metabolites (60%), or both (56%). The ASD group with very elevated arabinitol (17%) almost totally overlapped with the group with decreased N-formyl methionine (16%). All metabolite peak intensities were, on average, higher in the ASD group than in the TD group, except for N-formyl methionine, which had a significantly lower average value than the TD group.

MDM System^™^ Total Score: The MDM System^™^ Total Score is the number of MDMs higher than the concentration of any of the TD children. The ASD group had an average MDM Total Score of 3.3 MDMs, ranging from zero to nine, whereas the TD group all had an MDM Total Score of zero MDMs by definition. Ninety percent of the ASD participants (45 of 50) had one or more extremely elevated MDMs. By setting a cut-off for ASD of one or more extremely elevated MDMs, the MDM test yields a sensitivity of 90% and a specificity of 100%. All TD subjects are correctly classified, and 45 of the 50 ASD children are correctly classified.

Fisher Discriminant Analysis (FDA) demonstrated many combinations with high AUROC values:

Because the MDM System^™^ is a new multivariate evaluation approach, we used more traditional AI/ML techniques to compare against our novel methodology. Using Fisher Discriminant Analysis (FDA), combinations of 5 metabolites produced 1,014,572 unique combinations with AUROC > 0.8, with the highest reaching 0.86 after leave-one-out cross-validation (LOOCV). Neural Networks and Naïve Bayes approaches with cross-validation resulted in accuracies of 83% and 82%, respectively. Accuracies exceeding 80% are generally considered the target for these types of tests, and the approaches exceeded them. See supplemental table C and accompanying text for full details on multivariate analysis techniques and results.

### Quantitative-Targeted Metabolomics Analysis Confirmed Semiquantitative Measurements

Univariate Metabolite Results found several MDMs quantitatively measured significantly higher in ASD. Ten metabolites were significantly higher in the ASD group compared to the TD group, including 5 MDMs related to phenylalanine, four related to tryptophan, and one related to yeast (P-value between groups using Welsch’s test was less than 0.05, and no correction was performed for multiple hypothesis testing). The highest difference in average value between groups was 74,750% for tricarballylic acid, although it did not reach statistical significance, likely due to many values being below the limit of quantification.

For most metabolites, there was a small subgroup of ASD participants with concentrations of each MDM above that of any of the TD group, ranging from 0% to 21% (see Table 5), with the highest being 21% for *p*-cresol or *p*-cresol sulfate, a harmful metabolite that has been found elevated in ASD vs controls in 17 of 17 studies ^50^. The distribution of elevated metabolites is highly heterogeneous, with some children being unusually high in one metabolite and other children being unusually high in others. Several metabolites had a substantial number of participants below the limit of detection (see Table 5), so more sensitive methods are needed to fully investigate those metabolites.

Statistically significant results are noted in **BOLD** font. Phe/Tyr is the ratio of phenylalanine to tyrosine. DHPPA- Dihydroxyphenylpropionic acid. All values have units of micromoles per mole creatinine. Results are sorted by % of ASD above the TD reference range.

*P*-Cresol, *p*-cresol sulfate, indole-3-propionic acid, arabinitol, and phenylacetylglutamine showed the best inherent separation characteristics between groups due to a minimal correlation between metabolites. Among participants with autism spectrum disorder (ASD), 42% exhibited high levels of one or more tryptophan metabolites, 57% had elevated phenylalanine/tyrosine metabolites, and 26% had high levels of both one tryptophan metabolite and one phenylalanine metabolite. 16% of ASD participants showed increased levels of yeast metabolites.

Many MDMs were found to have high correlation with another. Table E in the supplement shows the correlation between concentrations of metabolites discovered during the analysis. Interestingly, indole propionic acid showed high correlations with several other metabolites, including an especially high correlation with beta-carboline. Beta carboline is a known metabolite of synthetic hallucinogenic compounds, but it is highly unlikely that any of the children in our sample groups had significant amounts of this compound through any means other than microbial dysbiosis^69,70^.

### The MDM System^™^ Results Generally Agreed Semi-quantitatively and Quantitatively

The MDM System^™^ found 78% sensitivity and 100% specificity following quantitative analysis. Semiquantitative results and quantitative results of the same samples yielded generally similar results. Table E (supplement) shows the data necessary to calculate positive predictive value, negative predictive value (sensitivity, specificity and overall accuracy of the MDM System^™^ for the untargeted and targeted data. The number of participants classified by the MDM System as either ASD positive or ASD negative compared to the diagnostic criteria established in the methodology section, including a physician’s diagnosis, CARS score, SRS-2 score, and PGIA score.

Additionally, FDA found 399 combinations of 3 metabolites with an AUROC of 0.7 or higher, with a maximum AUROC of 0.78, and combinations of 4, 5, or 6 metabolites only slightly increased the maximum AUROC to 0.80 (See supplement for detailed results) without cross-validation.

## Discussion:

Univariate analysis showed that participants with ASD have significantly higher peak intensities of many microbial metabolites compared to TD controls. Twenty-three of 24 microbially derived metabolites were statistically significantly higher in ASD than in TD participants. The metabolites were heterogeneously distributed, so that only a subset of ASD individuals had high concentrations of any individual MDMs, but most children with ASD had higher concentrations of one or more MDMs. The MDMs fell into three categories: Tyrosine/phenylalanine or tyrosine-derived, tryptophan-derived, and those created by yeast. Within each category, the compounds shared structural similarity, which suggests some degree of similar functionality. Some MDMs have a known toxic effect at high concentrations, and for others, their toxicity is unclear. Due to their close structural similarity to *p*-cresol and indoxyl sulfate, they are potentially harmful at high concentrations and may also contribute to ASD-related symptoms^50^. It is likely that the tryptophan-derived metabolites affect serotonin and melatonin-related neurotransmitter functions and that the phenylalanine-related metabolites affect dopamine-related neurotransmitter functions^71^.

To illustrate the potential neurological impact of these metabolites, we draw a parallel to ethanol, a well-known yeast-derived neurotoxin, which is commonly consumed by humans to affect their neurological function^72^. Ethanol effects at high concentrations—altered motor coordination, emotional dysregulation, slurred speech, and cognitive impairment—highlight how a single microbial metabolite produced by yeast can profoundly alter brain function. It is interesting to note that some other bacteria can sometimes produce ethanol^73^. Similarly, other microbial metabolites found elevated in ASD may contribute to neurological alterations such as sensory dysregulation, mood instability, and impaired language development.

The present results are qualitatively consistent with over 40 published results, finding high levels of MDMs in ASD ^1,10,15–18,25,36,37,43,45,48,50–52,54,55,57,60,63,64,66,74–95^, including p-cresol^36,45,50,76^, p-cresol sulfate^50^, indoxyl sulfate^52^ and phenylacetylglutamine^39^.

MDM metabolites contribute substantially to the overall ability of The MDM System^™^ to distinguish between ASD and controls. Some microbial metabolites like indole-3-acetonitrile and indole 3-acetoxidoximide have not previously been reported to be measured in ASD to the best of our knowledge, and this study found them to be significantly different in ASD. A total of 23 MDMs were statistically significantly higher in ASD in this study (See Tables 4 and 5).

### Semiquantitative Multivariate Analysis- The MDM System

The MDM System^™^ successfully classified children with ASD vs TD children with very high sensitivity and specificity. It builds on an extensive body of literature on elevated concentrations of certain MDMs in ASD (see Table 1).

The total score on the MDM system^™^ did not correlate significantly with age (data not shown), suggesting it may also be valid at younger and older ages, but more data with those populations is needed. For younger ages, the microbiome changes substantially when infants transition from breastmilk or formula to solid foods, so that may limit how early the MDM System^™^ can be used. New reference ranges may be needed for ages under 2 years. For ages above 11 years, the microbiome is similar to that of younger children, so the reference range may be similar for teens and adults. We are planning further investigations for teens and adults with ASD.

Of the fifty ASD participants in the semiquantitative analysis, five did not show elevated peak intensities of MDMs. Metabolomic analyses revealed that three of these participants likely have significant metabolic errors, potentially inborn, that may have underlain their ASD symptoms. One participant was suspected of having arginosuccinate lyase deficiency due to a large presence of arginosuccinate in the participant’s urine. Arginosuccinate lyase deficiency is a urea cycle disorder characterized by high concentrations of arginosuccinate and citrulline in blood or urine^96^. Another participant presented a metabolomic profile consistent with glutaric aciduria type II^97^, evidenced by high urinary concentrations of glutaric acid, lactate, hippuric acid, suberic acid, and adipic acid. A third participant exhibited a 100-fold increase in peak intensity of urinary propionic acid, suggesting a possible diagnosis of propionic or methylmalonic acidemia^98^. Each of these metabolic disorders has been associated with ASD or severe cognitive impairments and may present as either severe infantile forms or milder forms with later onset, which could potentially bypass newborn screening.

### Quantitative Analysis

During the semiquantitative analysis, 9 metabolites were confirmed as significantly higher in ASD. Four metabolites were below the limit of detection in a significant number of individuals, and thus were not able to be validated (See Table 4) and the footnote below,

### Multivariate Analysis

The MDM System^™^ resulted in 78% sensitivity and 100% specificity in the targeted analysis (See Fig. 1 and Table 5), demonstrating the algorithm’s value as a promising tool for early screening for ASD. The somewhat lower sensitivity than for the untargeted analysis is partly due to being unable to obtain some standards for the targeted analysis. When evaluating the overall accuracy of the MDM system^™^, it should be noted that some, but not all, of the participants overlapped between the quantitative and semiquantitative analyses.

### Limitations:

This study has several limitations. This study excluded participants with known single-gene disorders such as Fragile X, and the rate of extremely elevated MDMs in those groups is not known and requires further investigation. Although we have identified 24 MDMs, there are likely others that may also be increased in children with ASD. Finally, not all standards for all putatively identified metabolites were commercially available, such that the targeted quantitative analysis of some MDMs was not possible, thus reducing the sensitivity of the targeted vs the untargeted test.

## Conclusions

The major finding of this study is that approximately 8–9 of 10 young children with ASD in this cohort had high concentrations of one or more microbially-related metabolites. These results are consistent with over 40 studies that have measured concentrations of individual MDMs in ASD vs TD cohorts. Several of the MDMs have not previously been investigated in ASD, suggesting evidence of even more potential intestinal dysbiosis than previously recognized. The MDMs investigated fell into one of three categories: tryptophan-derived, phenylalanine (or tyrosine) derived, and yeast/fungi-derived. The former two result in MDMs that are likely to disrupt important neurotransmitter metabolism (serotonin, melatonin, and dopamine), which could adversely affect many ASD-related symptoms. Yeast-derived metabolites likely indicate an overgrowth in the GI tract, which may also contribute to ASD symptoms. MDMs can also impact many other parts of the body; for example, *p*-cresol is known to adversely affect the gut, brain, kidneys, liver, immune system and the mitochondria (which affects nearly every cell and organ in the body).

A major question is whether or not high concentrations of novel MDMs discovered in this analysis contribute to ASD symptoms. Some of the metabolites, like *p*-cresol and indoxyl sulfate, are known to cause ASD symptoms when administered to animals, and some studies have reported that *p*-cresol concentration correlates with worse ASD symptoms^25,35^. The other phenylalanine-derived and tryptophan-derived MDMs are similar in chemical structure to p-cresol and indoxyl sulfate, respectively, and thus, they share metabolic pathways, and it is possible that they may have similar adverse effects.

### Proposed New Phenotype of ASD

Based on our findings that a large subset of children with ASD in our multi-site cohort have elevated concentrations of one or more MDMs, we propose a new ASD phenotype, “ASD-associated with Microbially-Derived Metabolites “ (ASD-MDM), which is defined by quantitative laboratory measurements of MDMs in urine samples in young children. The development of the MDM System^™^ as a diagnostic screening tool for ASD is promising. Further validation of the MDM System^™^ in another cohort is underway by our research team, but the 40-plus studies of elevated concentrations of individual MDMs in ASD provide strong literature support for the MDM System^™^.

There is a critical need for a method to screen young children for ASD, since the current average age of diagnosis is 47 months in the US (for children diagnosed by age 8 years), and early intervention is much more effective if begun earlier in life. The MDM System^™^, based on a simple urine collection, is a unique non-invasive laboratory test that can provide diagnostic screening for the ASD-MDM phenotype, and the test is now available from Analutos (the lab that did the measurements for this paper) for samples sent to them from anywhere in the world. The lifetime cost of care for a person with ASD is estimated at $3.6 million, so we believe it is imperative to screen early in life to enable early intervention and better outcomes and quality of life..

Future research should focus on further development and further validation of the MDM System^™^, and treatment strategies aimed at re-establishing a healthy microbiome and thereby hopefully restoring normal gut-brain axis equilibrium. Methods such as Microbiota Transplant Therapy may hold promise as one study found that MTT substantially decreased p-cresol sulfate down to normal concentrations, along with substantially improving gut health and ASD-related symptoms^17^.

## Supplementary Material

Supplementary Files

This is a list of supplementary files associated with this preprint. Click to download.

• SupplementDocumentforMDMPaper.docx

## Figures and Tables

**Figure 2:**
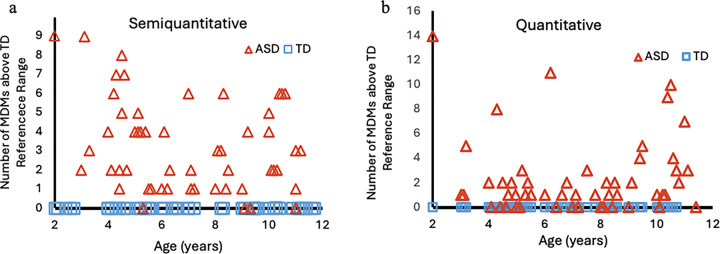
The MDM System^™^ Total Scores for Semiquantitative and Quantitative analysis. The vertical axis represents the number of microbially-derived metabolites above the reference range by individual, while the horizontal axis represents age. Note that all 47 TD individuals (Blue Squares) have a score of zero. a) Semiquantitative analysis- 45 of 50 ASD participants had at least one of the 17 microbial metabolites evaluated above the highest typically developing child (Range 1–9, average 3.91). b) Quantitative analysis- 40 of 52 ASD participants had at least one of the 23 metabolites evaluated above the highest typically developing child (Range 1–14, average 3.25).

**Table 1:** Urinary microbially-related metabolites reported as abnormal (almost always elevated) in ASD individuals compared to controls in other studies.

Metabolite	Relevant Findings
*p*-cresol/*p*-cresol sulfate	17 of 17 urinary metabolomics studies from 6 countries discovered the compound higher in ASD vs. TD^50^
Indoxyl sulfate	6 of 6 urinary metabolomics studies found the compound higher in ASD vs. TD^52^
Indoyl-3-acryloyl glycine	Higher in a subset of ASD patients^57^
Phenylacetylglutamine	Higher in a subset of ASD patients^39^
Arabinitol	Higher in a subset of ASD patients^54,55,58,59^
Hydroxybenzoic Acid	Higher in a subset of ASD patients^54,60^
Hippuric Acid	Higher in a subset of ASD patients^35,38,58,60^
Phenylalanine	Higher and or lower in a subset of ASD patients, directly involved in phenylketonuria, a known cause of ASD^61–63^
Benzoic Acid	Higher in a subset of ASD patient^54,55,58,60,64^
Citramalic Acid	Higher in a subset of ASD^35,54,55,59^
Tricarballylic Acid	Higher in a subset of ASD ^54,59,65,66^

**Table 2:** List of metabolites measured. Metabolites are arranged into three groups: phenylalanine-related metabolites, tryptophan-related metabolites, and yeast/other-related metabolites. Which metabolites were detected by Semiquantitative untargeted analysis undertaken by TGEN or quantitative targeted analysis by Analutos or both is indicated for each subsection.

**Phenylalanine Related Metabolites**
**Semiquantitative & Quantitative**
Phenylacetylglutamine
*p*-Cresol
*p*-Cresol Sulphate
**Semiquantitative**
4-(2-Aminopropoxy)-3,5-dimethylphenol
6 amino-m-cresol
4-Phenol Sulfonic Acid
**Quantitative**
Phenylacetic Acid
Phenyl propionic Acid
Phenylalanine/Tyrosine
Hydroxybenzoic acid
Hippuric Acid
Dihydroxyphenylpropionic acid (DHPPA)
Benzoic Acid
**Tryptophan Related Metabolites**
**Semiquantitative & Quantitative**
Indolyl-3-acryloylglycine (IAG)
4-Indolecarbaldehyde
3-Indolepropionicacid
1-Methyl-1,2,3,4-tetrahydro-Î^2^-carboline-3-carboxylic acid
5-Methoxy indole
*tans*-3-Indoleacrylic acid
**Phenylalanine Related Metabolites**
**Semiquantitative & Quantitative**
3-Indoleacetonitrile
Indoxyl Sulfate
**Semiquantitative**
3-Methyldioxyindole
5-Methoxyindole acetate
Methyl-3-Indole Acetate
Indole-3-acetaldoxime
**Yeast/ Other Microbial Metabolites**
**Semiquantitative & Quantitative**
Arabinitol
**Semiquantitative**
N-Formyl Methionine
**Quantitative**
Tricarballylic Acid
Tartaric Acid
Citramalic Acid

**Table 3:** Characteristics of Study Participants CARS-(Childhood Autism Rating Scale); SRS-2-(Social Responsiveness Scale 2nd edition); PGIA-(Parental Global Index); n.s.-Not Significant; n/a-not applicable

	ASD Group	TD Group	P-Value

Total Participants	52	47	n/a

Location:	28	23	n/a
Arizona	7	14	
Texas	11	11	
Tennessee	6	0	
Massachusetts			

Male	41	20	n/a
Female	11	27	
M:F ratio	3.5	0.74	

Average age (years)	6.7 ± 2.8	6.7 ± 3.0	n.s.

Cars Score	41 ±5.5	n/a	n/a

SRS-2 Score	114 ± 123.2	19.3 ± 10.8	< 0.001

PG.I.A. Score	2.8 ± 1.0	0.2 ± 0.32	< 0.001

**Table 4:** Phenylalanine/Tyrosine, Tryptophan, and Yeast-related microbial metabolites included in this analysis sorted by average difference between ASD and TD. The raw p-value, q-value, AUROC, and the percentage of ASD participants above the highest relative peak intensity for each metabolite is provided.

	Average Difference ASD vs. TD	Raw	q-Val	AUROC	% Above TD Range
		p-Val			
**Phenylalanine Related Metabolites:**					
4-(2-Aminopropoxy)-3,5-dimethylphenol	229%	**0.02**	0.04	0.70	10%
6-Amino-m-cresol	219%	**0.01**	0.01	0.66	12%
Phenylacetylglutamine	64%	**0.0001**	0.04	0.68	32%
p-Cresol	61%	**0.0001**	0.03	0.66	24%
p-Cresol Sulfate	54%	**0.002**	0.03	0.65	32%
4-phenol sulfonic acid	48%	**0.049**	0.03	0.67	4%
**Tryptophan Related Metabolites:**					
Methyl-3-Indole Acetate	1,882%	**0.009**	0.03	0.69	34%
3-Indolepropionic acid	561%	**0.0001**	0.04	0.71	34%
1-Methyl-1,2,3,4-tetrahydro-Î^2^-carboline-3-carboxylic acid	537%	**0.0009**	0.03	0.77	44%
3-Methyldioxyindole	125%	**0.002**	0.00	0.64	22%
5 Methoxy indole acetate	101%	**0.01**	0.038	0.69	12%
Indole-3-acryloyl glycine	98%	**0.02**	0.046	0.63	10%
*trans*-3-Indoleacrylic acid	98%	**0.004**	0.05	0.61	22%
4-Indolecarbaldehyde	57%	**0.03**	0.049	0.6	22%
3-Indoleacetonitrile	46%	**0.006**	0.049	0.65	14%
Indole-3-acetaldoxime	36%	**0.005**	0.03	0.64	14%
Indoxyl Sulfate	36%	**0.009**	0.04	0.67	0%
**Yeast Metabolites:**					
Arabinitol	51%	**0.002**	0.03	0.69	17%
N-Formyl Methionine	−71%	**0.05**	0.06	0.66	17%
**ASD Participants (%) with high tryptophan metabolites**					**64**
**Phenylalanine Related Metabolites:**					
**ASD Participants (%) with high phenylalanine metabolites**					**60**
**ASD Participants (%) with high yeast metabolites**					**17**

**Table 4:** Quantitative Results for phenylalanine-related, tryptophan-related, and yeast/other metabolites. All MDM metabolite levels are umol per mmol creatinine (umol/mmolCr), except for aromatic amino acids, Phenylalanine (Phe) and Tyrosine (Tyr) which are in nanograms per mole Creatinine (nmol/molCr). The mean values for the ASD group, the TD group, as well as the mean difference and statistical significance, the percent of ASD participants with higher levels than any TD participant, and what proportion of overall participants had levels below the limit of detection.

Phenylalanine Based Metabolites						
	Mean ASD	Mean TD	% Difference	p-value	Percent of ASD above TD Range	% Below LOD
*p*-Cresol Sulphate	2564	1072	139%	**0.02**	21%	0%
*p*-Cresol	0.44	0.25	76%	**0.003**	19%	0%
Hydroxybenzoic acid	4.73	1.03	360%	**0.01**	17%	31%
Benzoic Acid	1.26	0.71	77%	**0.04**	13%	8%
Phenylacetylglutamine	0.80	0.44	80%	**0.002**	13%	0%
Phe/Tyr	1.65	1.22	36%	0.31	10%	0%
Benzoic/hippuric	0.02	0.01	22%	0.83	8%	3%
Hippuric Acid	84	54	55%	**0.04**	8%	0%
DHPPA	0.64	0.56	14%	0.66	4%	8%
Phenylacetic Acid	1.50	1.17	28%	0.26	4%	15%
Phenyl propionic Acid	0.01	0.03	−47%	0.68	0%	76%
Tryptophan Based Metabolites						
	Mean ASD	Mean TD	% Difference	p-value	Percent of ASD above TD Range	Below LOD
Indole-3-acryloyl glycine	7.95	3.77	111%	**0.03**	17%	0%
3-Indolepropionic acid	0.02	0.01	78%	0.61	15%	0%
4-Indolecarbaldehyde	0.02	0.01	319%	0.48	13%	0%
3-Methyldioxyindole	0.00	0.00	497%	0.32	12%	70%
Indoxyl Sulfate	43	16	171%	**0.03**	10%	0%
2-oxindole	0.00	0.00	3,015%	0.32	8%	94%
3-Indoleacetonitrile	0.00	0.00	103%	0.33	6%	8%
1-methyl-1,2,3,4 Tetrahydro Beta Carboline	0.022	0.008	183%	0.57	6%	0%
5-methoxyindole	0.000	0.000	−95%	0.33	0%	95%
Yeast Metabolites:						
	Mean ASD	Mean TD	% Difference	p-value	Percent of ASD above TD Range	Below LOD
Arabinitol	37	27	41%	**0.01**	10%	0%
Citramalic Acid	0.11	0.06	85%	0.47	4%	0%
Tartaric Acid	0.274	0.053	413%	0.69	2%	36%
Tricarballylic Acid	0.845	0.001	74,570%	0.35	0%	30%
**ASD Participants (%) with high tryptophan metabolites**						**42**
**ASD Participants (%) with high phenylalanine metabolites**						**57**
**ASD Participants (%) with high yeast metabolites**						**16**
**ASD Participants (%) with any high MDM metabolites**						**78**
